# *var *gene transcription and PfEMP1 expression in the rosetting and cytoadhesive *Plasmodium falciparum *clone FCR3S1.2

**DOI:** 10.1186/1475-2875-10-17

**Published:** 2011-01-25

**Authors:** Letusa Albrecht, Kirsten Moll, Karin Blomqvist, Johan Normark, Qijun Chen, Mats Wahlgren

**Affiliations:** 1 Department of Microbiology, Tumor and Cell Biology (MTC), Karolinska Institutet, Box 280, SE-171 77 Stockholm, Sweden; 2 Department of Molecular Biology, Umeå University, Umeå SE-90187, Sweden; 3 Chinese Academy of Medical Sciences, Beijing, China

## Abstract

**Background:**

The pathogenicity of *Plasmodium falciparum *is in part due to the ability of the parasitized red blood cell (pRBC) to adhere to intra-vascular host cell receptors and serum-proteins. Binding of the pRBC is mediated by *Plasmodium falciparum *erythrocyte membrane protein 1 (PfEMP1), a large multi-variant molecule encoded by a family of ≈60 *var *genes.

**Methods:**

The study of *var *gene transcription in the parasite clone FCR3S1.2 was performed by semi-quantitative PCR and quantitative PCR (qPCR). The expression of the major PfEMP1 in FCR3S1.2 pRBC was analysed with polyclonal sera in rosette disruption assays and immunofluorecence.

**Results:**

Transcripts from *var*1 (FCR3S1.2_*var*__1_; IT4*var*21) and other *var *genes were detected by semi-quantitative PCR but results from qPCR showed that one *var *gene transcript dominated over the others (FCR3S1.2_*var*__2_; IT4*var*60). Antibodies raised in rats to the recombinant NTS-DBL1α of *var*2 produced in *E. coli *completely and dose-dependently disrupted rosettes (≈95% at a dilution of 1/5). The sera reacted with the Maurer's clefts in trophozoite stages (IFA) and to the infected erythrocyte surface (FACS) indicating that FCR3S1.2_*var2 *_encodes the dominant PfEMP1 expressed in this parasite.

**Conclusion:**

The major transcript in the rosetting model parasite FCR3S1.2 is FCR3S1.2_*var*__2 _(IT4*var*60). The results suggest that this gene encodes the PfEMP1-species responsible for the rosetting phenotype of this parasite. The activity of previously raised antibodies to the NTS-DBL1α of FCR3S1.2_*var*__1 _is likely due to cross-reactivity with NTS-DBL1α of the *var*2 encoded PfEMP1.

## Background

The malaria parasite *Plasmodium falciparum *causes the death of around one million individuals annually, mainly small children. There are an estimated 300 million clinical cases annually in the world despite the fact that individuals are able to acquire immunity to the disease [1]. Protective immunity towards malaria develops, however, only after repeated exposure to the *P. falciparum *parasite and it is known to be in part dependent on antibodies towards the variable antigens present at the pRBC surface [2-9]. The best-characterized molecule of these surface antigens is the *P. falciparum*-infected erythrocyte membrane protein 1 (PfEMP1). This protein family is encoded by a repertoire of around 60 *var*-genes per genome and the parasite can switch between different variants that are exported to the surface of the pRBC in order to evade the host's immune system [10]. In addition, the molecule PfEMP1 plays a central role in the parasite's ability to sequester in the microvasculature of the infected individual and to form rosettes between infected and uninfected RBC as well as giant-rosettes or auto-agglutinates [3,11,12]. Since PfEMP1 can bind to a variety of host-cell receptors the pRBC is able to avoid clearance in the spleen thus contributing substantially to the manifestations of severe malaria through excessive sequestration [13].

**Figure 1:**
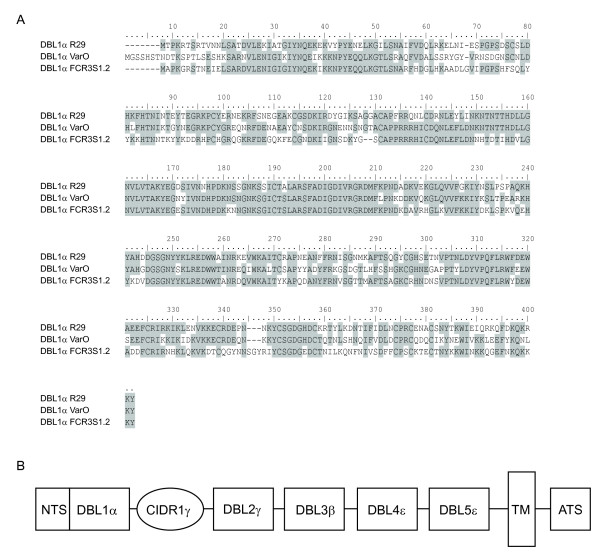
**Structure and sequence comparison of the FCR3S1.2**_***var***__**2 **_**gene **A: Alignment of the protein sequence of the rosette associated DBL1α-domains of the parasite strains FCR3S1.2, R29 and Palo Alto varO. B: Schematical presentation of the FCR3S1.2_*var*__2 _gene structure.

The N-terminal Duffy-binding like domain (DBL) 1α has the highest degree of sequence conservation among all domains of PfEMP1 [14,15] and it is responsible for binding to host receptors both on RBC and on endothelial cells [16-18]. This domain has, therefore, a central role in parasite sequestration in the microvasculature [18-20] and certain characteristics have been associated with severe disease [5,12,21-24]. DBL1α-domains of PfEMP1 of parasites of rosetting phenotypes have been described for the strains R29 [16], varO [25] and the clone FCR3S1.2 [17,26] (sequence alignment compare Figure 1A) which is the focus of this article.

*var *genes can be divided into five different classes according to their 5' upstream region [27] and it has been found, that rosetting parasites more frequently express *var *genes belonging to group A, and group A/B are more often transcribed in patients suffering from severe malaria [12,28-30]. The transcribed *var *gene repertoire in the rosetting parasite strain FCR3S1.2 was recently re-analysed and found that the dominant transcript is FCR3S1.2_*var2 *_(IT4*var*60) (Figure 1B) that also belongs to the group A-*var *genes. The original analysis and identification of the FCR3S1.2_*var1 *_as the dominant transcript [26] was carried out using degenerated primers modified after Su *et al *[31]. Now optimized RNA extraction and RT-PCR protocols were applied [12,32] using three sets of primer-pairs generated for the amplification of unknown DBL1α-sequences [12]. These transcripts were subsequently amplified using qPCR. With this approach, a different *var *gene (FCR3S1.2_*var2*_) was found to be the dominantly transcribed in FCR3S1.2. Sera raised against the NTS-DBL1α-domain of FCR3S1.2_*var*__2 _showed a PfEMP1-specific immunofluorescence pattern, stained the FCR3S1.2 pRBC surface in FACS and disrupted the rosettes of this parasite clone.

## Methods

### Parasite cultures

The *P. falciparum *laboratory clone FCR3S1.2 [33] was cultivated as described in blood group O RBC [34]. The phenotype of FCR3S1.2 was maintained by weekly enrichment over a Ficoll-gradient [34]. FCR3S1.2 pRBC of different generations after cloning were used in the experiments. Transcription levels were measured in different cultures of FCR3S1.2 including: A) parasites 18 generations after micromanipulation cloning (FCR3S1.2-18G; 85% rosetting) [33]; B) parasites ≥100 generations after cloning (FCR3S1.2-100 G, 85% rosetting) and C) parasites after 28 generations of additional growth of the parasites of (B) (FCR3S1.2-100 G) in the absence of Ficoll enrichment (FCR3S1.2 128 G, 78% rosetting). The pRBC of FCR3S1.2 avidly bind to a number human cellular receptors and serum-proteins (Table 1).

**Table 1:** Summary of adhesive characteristics of pRBC of the *P. falciparum *clone FCR3S1.2

*in vivo*	
Intravascular sequestration in Sprague Dawley rats^a^	+
Intravascular sequestration in *Macaca fascicularis*^a^	+
***in vitro***	

Rosetting (% rosetting pRBC)^b^	80-90%
Giant-rosetting/auto-agglutination^b^	+
Soluble heparin^c^	90%
Blood Group A^c^	90%
Ig-binding anti-Ig^c^	96%
Ig-binding anti-IgM^c^	90%
Ig-binding anti-IgG^c^	12-20%
HUVEC^d^	1,200-1,600
Melanoma cells^d^	400-500
CHO-CD36^d^	200-300
CHO-ICAM1^d^	40 ± 12
CHO cells^d^	6 ± 3
L-cells (PECAM-1/CD31)^d^	390 ± 28
L-cells^d^	5
sPECAM-1/CD31^d^	183 ± 31
TSP^e^	-
CSA^e^	-
Placenta^f^	0

a) Rats or macaques were administrated with ^99 m^Technetium-labeled pRBC of the FCR3S1.2 clone by injection into the tail vein (rats) or *Vena saphena magna *(macaques). The animals were left for 60 min after which sequestration was measured in a triple headed-gamma camera [20,44]

b) Rosetting rates are expressed as the range of percent rosetting trophozoite-pRBC [33].

c) Percentage of late stage pRBC showing surface fluorescence when incubated with antibodies to human non-immune Ig, IgM or IgG, or heparin, or the blood group ABO antigens [33]

d) Number of pRBC bound per 100 cells [17,33]

e) Number of late stage pRBCs bound to thrombospondin-coated plastic (50 mg/ml)

f) Number of IEs bound to 1 mm^2 ^of placental tissue

### RNA extraction, amplification of *var *transcript by RT-PCR and semi-quantitative PCR

RNA was extracted from FCR3S1.2 early trophozoites (18-24 h p.i.) at ≥100 generations after cloning with the QiagenRNeasy kit with minor modifications, followed by treatment with TURBO DNAse in order to remove any remaining DNA (Ambion, Austin TX, USA) as previously described [32]. Reverse transcription was carried out using superscript III (Invitrogen, Carlsbad, CA, USA) with random hexamers and oligo(dT)12-18 (300 ng/ml and 25 ng/ml respectively) at 25°C for 10 min and 50°C for 120 min followed by 70°C for 15 min. A control reaction without reverse transcriptase (RT-) was performed for each cDNA synthesis reaction. cDNA was used as template for PCR with the degenerated primers nDBLf (TKGCAGCMAAWTAYGARGX), nDBLr (KTCCACCAATCTTCYCT), α-AF (GCACGMAGTTTTGC) and α-BR (GCCCATTCSTCGAACCA). AccuTaq LA DNA polymerase mix was used (Sigma, Saint Louis, MA, USA) and the cycling conditions were 3 min denaturation followed by 35 cycles of 30 sec at 45°C, 45 sec at 60°C, 15 sec at 94°C and finally 7 min at 72°C [12,32]. PCR products were cloned using the TOPO TA cloning kit (Invitrogen). Forty-eight clones for each primer pair were subsequently sequenced using the MegaBace system.

### Quantitative PCR

In order to quantify the transcripts identified by semi-quantitative PCR in FCR3S1.2 quantitative PCR (qPCR) was performed as previously described [32]. Oligonucleotides were designed based on DBL1α sequences obtained from the semi-quantitive PCR, using Primer Express (version 3.0, Applied 215 Biosystems, Foster City, CA, USA) and Netprimer (Premier Biosoft, 216 Palo Alto, CA, USA) (Additional file 1).

The evaluation of *var *transcripts was done for pRBC of A) parasites 18 generations after cloning (FCR3S1.2-18 G, 85% rosetting), B) parasites ≥100 generations after cloning (FCR3S1.2-100 G, 85% rosetting) and C) with pRBC of FCR3S1.2 grown for an additional 28 generations without any Ficoll enrichment (FCR3S1.2-128 G, 78% rosetting). After RNA extraction and DNAse treatment (as described above) the RNA was analysed using 2100 Bioanalyzer (Agilent Tecnology). The RNA was further processed when the RNA integrity (RIN) was above 5 [35]. qPCR reactions were prepared in quadruplicates containing Power SYBR Green master mix (Applied Biosystems) in 10 μl volumes at a 300 nM concentration for each primer. Quantitative amplifications were performed through 45 cycles (95°C for 15 s and 60°C for 1 min) in an ABI 7900 qPCR system (Applied Biosystems). Seryl-tRNA-synthetase was used as an endogenous control for relative quantification. qPCR results were analysed as earlier described [32].

### Production of NTS-DBL1α_var2 _protein in *E. coli *and polyclonal NTS-DBL1α_var2 _sera

The NTS-DBL1α_var2 _domain was amplified from genomic DNA of the parasite clone FCR3S1.2 (forward primer: *CCA TGG *CAC CAA AGG GTA GAA; reverse primer: *AGA TCT *GTA TTT TTTTTT TTG TTT ATT AAA TTC) and cloned into the pQE60 vector (vector pQE, Qiagen, USA), expression and purification of his-tagged NTS-DBL1α_var2 _domain was carried out as described [36]. Three male Sprague Dawley rats (3 months old) received four immunizations with recombinant NTS-DBL1α_var2 _protein on days 0, 30, 60 and 90 (50 μg/rat) emulsified in Freund's complete (first immunization) or incomplete adjuvant (second to fourth immunization). Sera were collected four weeks after the last immunization.

### Rosette disruption assay

The capacity of the immune sera raised in rats against recombinant NTS-DBL1α_var2 _to disrupt rosettes of the FCR3S1.2 clone in blood group O RBC (dilutions: 1:5, 1:10, 1:20, 1:40, 1:80) was assayed as described [17]. As a positive control a purified IgG fraction of a Malawian hyper-immune sera pool was used. Malaria naïve Swedish sera and pre-immune rat sera were used as controls.

### Immunofluorescence assays with anti NTS-DBL1α_*var2*_sera

Air-dried monolayers of trophozoite-pRBC of the FCR3S1.2 clone were obtained as described previously [37]. The monolayers were incubated 60 min with polyclonal NTS-DBL1α_var2 _sera diluted in PBS (1:50), washed three times in PBS, and incubated 60 min with an ALEXA488- conjugated goat anti-rat antibody (Molecular Probes, Invitrogen). All incubations were carried out at RT in a humid chamber. Preparations were mounted with an anti-fading solution consisting of 20% DABCO (Sigma) in glycerol, and analysed with 10× ocular and a 100× oil immersion lens in a Nikon Eclipse 80i microscope.

### Analysis of surface recognition by flow cytometry

Trophozoite pRBCs of ≈24-30 p.i. were incubated in a dilution of 1:20 with the polyclonal NTS-DBL1α_var2 _rat sera for 30 min at RT. The pRBC were washed twice with PBS/FCS after incubation with the primary antibody followed by a 30 min incubation with a goat anti-rat IgG antibody coupled to ALEXA488 (dilution 1:100). For nuclear staining ethidium bromide was added at final concentration 2.5 μg/ml and resuspended in PBS with 2% FCS. The cell acquisition was done using flow cytometry (FACSCalibur, BD Bioscience, http://www.bd.com) where 5000 pRBC were counted. The analysis was performed using the software Cell Quest Pro (BD Bioscience).

## Results

### Amplification of the transcribed *var *gene in FCR3S1.2 by RT-PCR

**Figure 2:**
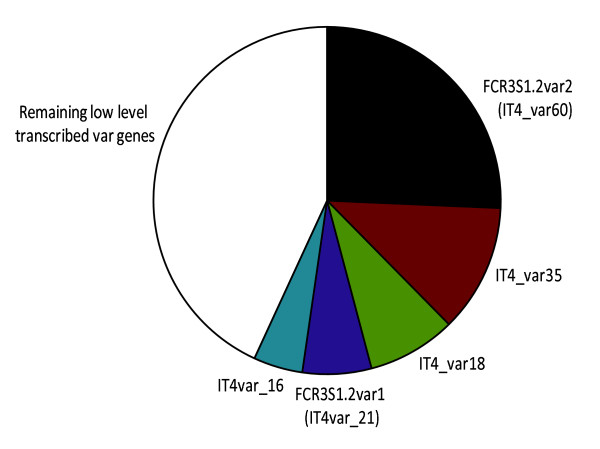
***var *gene transcription in FCR3S1.2 trophozoites by semi-quantitative PCR**. The pie slices depict the total relative distribution of each *var *gene amplified with three different degenerate primer pairs. The five most frequently transcribed *var *genes are shown. The white field indicates the remaining low level transcribed *var *genes.

The reverse transcriptase-PCR (RT-PCR) amplification was carried out with three sets of degenerate primer pairs (α-AF/α-BR, nDBLf/nDBLr, nDBLf/α-BR) and sequencing generated a total of 111 sequence reads after post quality control. These were divided into contigs and analysed as previously described [12,32]. FCR3S1.2 expressed a range of *var *genes, not necessarily full-length transcripts; in total 40 *var *genes were amplified, where five of those sequences were present in 60% of the total reads (Figure 2). A gene referred to as *var2 *(IT4var60) was the most dominant *var *gene transcribed with 28% of the reads. Low transcript levels of the *var*1 gene were also detected (6.3% of the reads). The distribution of transcribed *var *genes is shown in Figure 2.

### Amplification of the transcribed *var *gene in FCR3S1.2 by qPCR

**Figure 3:**
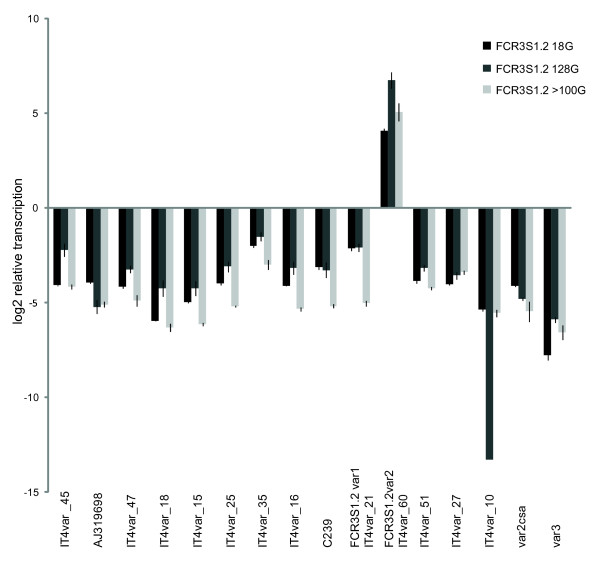
**Relative *var *transcript levels in the pRBC of three distinct FCR3S1.2 cultures as seen by qPCR**. The most frequently identified FCR3S1.2 *var *transcripts were amplified and the levels were compared to that of the endogenous control, *Seryl-tRNA-synthetase*. The *var *genes amplified had previously been identified by semi-quantitative PCR and sequencing by employing three sets of degenerate primers to the 5-prime end of the *var *genes[12]. Transcription levels were measured in different cultures of FCR3S1.2. A) 18 generations after micro-manipulation cloning (FCR3S1.2 18 G; 85% rosetting; [33], B) 100 generations after cloning (FCR3S1.2 >100 G, enrichment on Ficoll to maintain rosetting, 85% rosetting), and C) at 28 generations of additional growth of FCR3S1.2 100 G without Ficoll enrichment (FCR3S1.2 128 G, 78% rosetting). Results are visualized as log2 transformed values.

After the identification of *var *genes transcripts by semi-quantitative PCR a depth analysis was performed using qPCR. The five most frequent *var *transcripts but also nine minor transcripts identified in the semi-quantitative PCR and two conserved *var *transcripts (*var2csa *and *var3*) were analysed by qPCR. The predominant *var *gene identified here was identical with the one found by semi-quantitative PCR, here named FCR3S1.2_*var*__2 _(Figure 3). Independent of the number of generations after cloning (18 or ≥100 generations) or minor differences in the rosetting rate (85% versus 78%), the results showed the same dominant *var *transcript. This transcript has 100% identity with the DBL1α domain of IT4*var*60 [15]. The semi-quantitative PCR and qPCR results, therefore, suggest that FCR3S1.2_*var*__2 _is the most dominant *var *transcript in FCR3S1.2. Minor transcripts, such as IT4*var*21 (*var*1) and IT4*var*35 were also found employing qPCR, in particular in the earlier generations of the FCR3S1.2 parasite clone (FCR3S1.2 18 G).

### Analysis of the expressed PfEMP1 in FCR3S1.2

**Figure 4:**
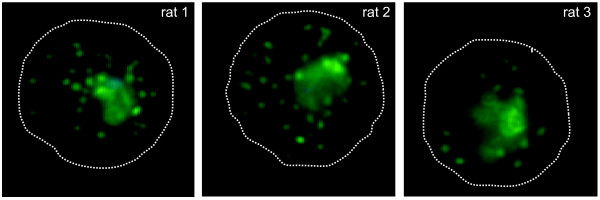
**PfEMP1 of FCR3S1.2_var2_**. Indirect staining of pRBC of FCR3S1.2 with anti-NTS-DBL1α_var2 _sera from three individual rats: Maurer's-cleft pattern is observed when staining air-dried monolayers of pRBC with anti-NTS-DBL1α_var2 _sera (green). Parasite nuclei were counterstained with Hoechst (blue). Pre-immune sera did not show any reactivity.

**Figure 5:**
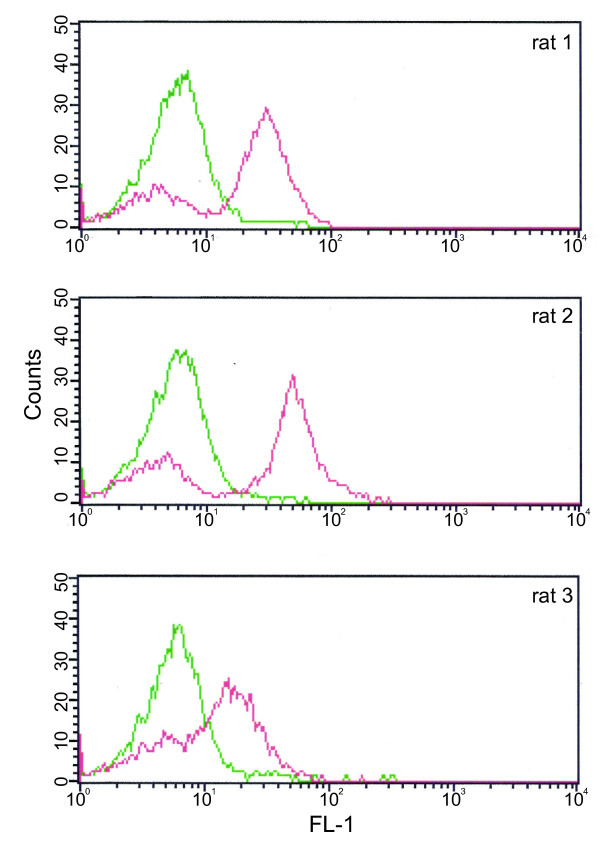
**Surface staining of live pRBC of FCR3S1.2**. Sera generated by immunizing rats with NTS-DBL1α_var2 _were studied in flow cytometry for their ability to react with the pRBC surface. The staining obtained with the pre-immune serum is shown in green, the immune sera in red. The figure shows the results obtained with sera of three rats (rat 1-3).

The expression of PfEMP1 in the parasite clone FCR3S1.2 was analysed using sera raised in rats against the *Escherichia coli*-produced, his-tagged NTS-DBL1α_var2_. Indirect immunofluorescence was carried out on air-dried monolayers of pRBC harbouring trophozoite stages of 24-30 h p.i.. The observed pattern in the pRBC showed staining of multiple vesicular structures typical for trafficking of PfEMP1 in Maurer's clefts (Figure 4). Pre-immune sera from the same animals did not show any staining of the pRBC-monolayers. In addition, staining of live pRBC with the anti-NTS-DBL1α_var2 _was carried out using flow-cytometry and IFA of live pRBC. Around 75% of the pRBC showed surface reactivity with the anti-NTS-DBL1α_var2 _sera (Figure 5). The number of pRBC showing surface reactivity correlated to the rate of FCR3S1.2 pRBC involved in rosetting/auto-agglutination in each experiment. Pre-immune sera of the immunized rats did not result in any surface reactivity with the pRBC.

### Functional analysis of anti-NTS-DBL1α_var2 _antibodies

**Figure 6:**
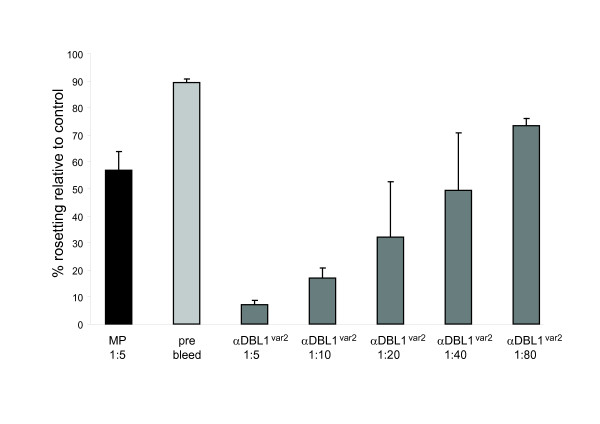
**Disruption of rosettes formed by pRBC of FCR3S1.2**. Sera raised in rats against NTS-DBL1α_var2 _were assayed in different dilutions for their capacity to disrupt rosettes and auto-agglutinates. A pre-immune rat serum was used as a negative control (pre-bleed) and a pool of human hyper-immune IgG obtained from Malawi was used as a positive control (MP). The rosetting rate is expressed as the relative rosetting rate compared to the untreated control.

Functional analysis of the anti-NTS-DBL1α_var2 _antibodies was carried out by analyzing their capacity to disrupt rosettes and auto-agglutinates of the clone FCR3S1.2. The sera were assayed in duplicates in dilution series from 1:5 to 1:80 and showed a strong and dose-dependent ability to disrupt rosettes as compared to a non-treated control (relative rosetting rates: 7.2% for dilution 1:5, 17.1% for 1:10, 32.1% for 1:20, 49.4% for 1:40, 73.5% for 1:80 and 89.3% for pre immune serum; Figure 6). The effect of the anti-NTS-DBL1α_var2 _serum on rosetting in FCR3S1.2 was higher than that of a hyper-immune pool from Malawi, which in a dilution of 1:10 gave a relative rosetting rate of 56.9% as compared to 10.1% for the anti-NTS-DBL1α_var2 _serum. Pre-immune sera from the same animals did not disrupt rosettes and auto-agglutinates.

## Discussion

Here, the FCR3S1.2_*var*__2 _gene coding for the PfEMP1 that mediates rosetting in the parasite strain FCR3S1.2 was identified. Furthermore, it was shown that antibodies towards the NTS-DBL1α-domain of FCR3S1.2_*var*__2 _recognize the surface of the pRBC and are able to disrupt rosettes of FCR3S1.2 parasite.

New tools have been developed for the study of molecular processes and their quality has improved over the last 15 years. Further, the accessibility of complete *P. falciparum *genomes [15,38] and a number of studies analysing variable sequences has facilitated the study of polymorphic genes in this pathogen [12]. Sequence information has helped in the development of ways to analyse the variable genes. In 1998 when the dominant *var *transcript in the rosetting parasite FCR3S1.2 was first identified [26], only few *var *sequences were available and the tools for studying these genes were limited. At that time, the single cell PCR and Northern blot techniques were used to recognize the dominant transcript in FCR3S1.2 leading to the identification of the gene FCR3S1.2_*var*__1 _[26]. In contrast, in the present study, two different primer pairs in three different combinations in RT-PCR were applied [12,32]. Thereafter and based on the results from the RT-PCR, primers to the five most dominant *var *genes as well as to two conserved *var *genes (*var3 *and *var2CSA*) were designed. In addition, nine *var *genes that were found as minor transcript (less than 4 sequence-reads) were included. These primers were subsequently used to analyse the *var *gene transcription by quantitative PCR. Using this more in-depth approach we found a different FCR3S1.2 *var *gene (*var*2) to be predominantly transcribed in the same parasite.

**Figure 7:**
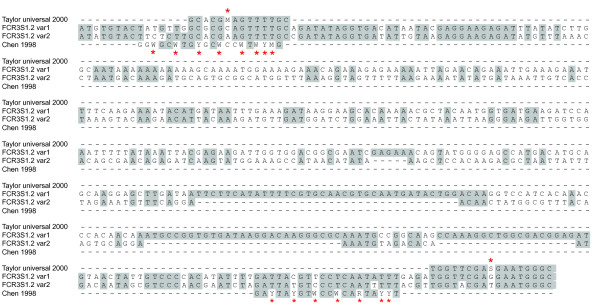
**Sequence alignment of the DBL1α-domain sequence and PCR-primers used **Comparison of the DBL1α- and oligonucleotide-sequences to depict mismatches of the primers used in the original identification of the dominant *var *gene in the FCR3S1.2 parasite: Aligned are the "Taylor universal 2000" [43] oligonucletides, the DBL1α-sequence of *var*1, *var*2 of FCR3S1.2 as well as the originally used oligonucleotides named "Chen 1998" [26]. The stars indicate degenerate nucleotides in the primer pairs.

In order to investigate whether a *var *gene switch had occurred during growth of the parasite over the years, parasites frozen immediately after the cloning of the FCR3S1.2 (18 generations) were compared to more than 100 generations after cloning. In both cases, the same predominant *var *transcript was found, suggesting that the *var *gene FCR3S1.2_*var*__2 _(IT4*var*60) was already the major transcript at the time of cloning. The primer pair used in 1998 for the identification of FCR3S1.2_*var*__1 _transcript in FCR3S1.2 includes several degenerated nucleotides (8/20 in the forward primer and 7/20 in the reverse primer) (Figure 7). This could be the reason why *var*1 was identified, although this primer pair shows no bias [26].

Functional assays with polyclonal antibodies towards the NTS-DBL1α_var2 _show that these antibodies are able to disrupt almost 100% of the rosettes in the homologous parasite whereas antibodies against the NTS-DBL1α_var1 _disrupt rosettes to a lower extent in the same parasite (Additional file 2) [19,39]. Cross-reactivity of NTS-DBL1α_var1 _antibodies to heterologous NTS-DBL1α-domains such as the one of the R29 parasite strain has also previously been reported by this group [39]. In addition, the ability of antibodies to disrupt rosettes and impair sequestration in the rat has been observed in several other studies suggesting that anti-PfEMP1 antibodies may cross-react in-between different parasites [4,5].

Depending on the 5' upstream region, *var *genes can be divided into 5 different subgroups, the FCR3S1.2_*var*__2 _also known as IT4*var*60 gene identified here belongs to the group A *var *genes [15]. FCR3S1.2_*var*__2 _lacks two cysteines in the DBL1α domain as compared to FCR3S1.2_*var1 *_and this characteristic has previously been associated with a rosetting phenotype and/or severe malaria [12,25,40,41]. The FCR3S1.2_*var2*_/IT4*var*60 gene was also found associated with the rosetting phenotype in a previous study [42]. Taken together this suggests that DBL1α_var2 _of FCR3S1.2 has characteristics that fit well with the rosetting phenotype of this parasite. Still, the results do not entirely discard the possibility that a small population of the FCR3S1.2 parasites transcribes the previously identified FCR3S1.2_*var*__1_. The rosetting rate when enriched on a Ficoll gradient, is about 85-90%, but never reaches 100%, a fact which may indicate that a small subpopulation of the parasites express PfEMP1 variants other than the PfEMP1 encoded by *var*2.

## Conclusions

The results presented here show that the FCR3S1.2 parasite clone expresses one dominant *var *gene transcript, FCR3S1.2_*var*__2 _(IT4*var*60), which belongs to the group A *var *genes. The encoded PfEMP1_var2 _carries a two-cysteine-signature associated with rosetting and antibodies to the protein avidly stain the pRBC suggesting that FCR3S1.2_*var*__2 _is the dominant *var *gene expressed in this parasite.

## Abbreviations

DBL: Duffy-binding like; PfEMP1: *Plasmodium falciparum *erythrocyte membrane protein 1; pRBC: parasitized red blood cell; qPCR: quantitative PCR

## Competing interests

The authors declare that they have no competing interests.

## Authors' contributions

LA carried out qPCR experiments and analysis. KM generated recombinant protein, sera and carried out IFA and rosette disruption experiments. KB and JN carried out semi-quantitative PCR and designed qPCR primers. LA, KM, KB, MW and QC drafted and wrote the manuscript. All authors have read and approved the final manuscript.

## Supplementary Material

Additional file 1 **Oligonucleotides used for qPCR experiments**. The data provided show the oligonucleotides used for qPCR are described below or previously described by Blomqvist *et al *[32]. Click here for file

Additional file 2 **Rosette disruption in FCR3S1.2 pRBC**. Rosette disruption of FCR3S1.2 pRBC with sera raised against the DBL1α-domain of FCR3S1.2 _*var*__1_, respectively FCR3S1.2_*var*__2_ Click here for file
