# Supplementary material for: var gene transcription and PfEMP1 expression in the rosetting and cytoadhesive Plasmodium falciparum clone FCR3S1.2
Source: Malar J. 2011 Jan 25;10:17. doi: 10.1186/1475-2875-10-17 (PMC3036667; doi:10.1186/1475-2875-10-17)
Supplement: Additional file 1 — Oligonucleotides used for qPCR experiments. The data provided show the oligonucleotides used for qPCR are described below or previously described by Blomqvist et al [32]. [file 1475-2875-10-17-S1.DOC]

**Additional file 1**

Oligonucleotides used for qPCR are described below or previously described by Blomqvist *et al*. (32).

| IT4var60 (var2) forward | TTCAGGAACAACTATGGCGTTAC |
| --- | --- |
| IT4var60 (var2) reverse | TGGGACGCTATTGTCATTGTG |
| IT4var45 forward | TCGAGCAACATGTGGTGATAGTG |
| IT4var45 reverse | CTTGGTCGCCGTCCTTTG |
| C239 forward | TGGAGCCGAAGCTAATCACA |
| C239 reverse | TCATTGGACTTGTTGTCTCTTGACT |
| IT4var15 forward | AGACAAACAGTATGGAAAGCTATCACA |
| IT4var15 reverse | ATTAGTCCACGTTCCTGTACCACAT |
| IT4var21 (var1) forward | AACAGTATGGGAAGCCATGACA |
| IT4var21 (var1) reverse | CACGTTGCACGAAAATATGAAGA |
| IT4var35 forward | TAAAGTATGGGAAGCTATAACATGTGATG |
| IT4var35 reverse | CCTTGTTTATGGCCGCATTTAG |
