# Supplementary material for: var gene transcription and PfEMP1 expression in the rosetting and cytoadhesive Plasmodium falciparum clone FCR3S1.2
Source: Malar J. 2011 Jan 25;10:17. doi: 10.1186/1475-2875-10-17 (PMC3036667; doi:10.1186/1475-2875-10-17)
Supplement: Additional file 2 — Rosette disruption in FCR3S1.2 pRBC. Rosette disruption of FCR3S1.2 pRBC with sera raised against the DBL1α-domain of FCR3S1.2 var1, respectively FCR3S1.2var2 [file 1475-2875-10-17-S2.DOC]

**Additional file 2**

Rosette disruption of FCR3S1.2 pRBC with sera raised against the DBL1α-domain of FCR3S1.2*_var_*_1_, respectively FCR3S1.2*_var_*_2_

|  | dilution of sera | % rosette disruption |
| --- | --- | --- |
|  |  |  |
| α-var1 sera | 1:5 | ≈20 |
|  | 1:10 | ≈15 |
|  | 1:20 | ≈5 |
|  |  |  |
| α-var2 sera | 1:5 | ≈90 |
|  | 1:10 | ≈80 |
|  | 1:20 | ≈70 |
|  |  |  |
